# Ras inhibition boosts galectin-7 at the expense of galectin-1 to sensitize cells to apoptosis

**DOI:** 10.18632/oncotarget.844

**Published:** 2013-02-24

**Authors:** Batya Barkan, Adrienne D. Cox, Yoel Kloog

**Affiliations:** ^1^ Department of Neurobiology, The George S. Wise Faculty of Life Sciences, Tel Aviv University, Tel Aviv, Israel; ^2^ Departments of Radiation Oncology and Pharmacology, Lineberger Comprehensive Cancer Center, University of North Carolina at Chapel Hill, Chapel Hill, USA

**Keywords:** apoptosis, c-jun, farnesylthiosalicylic acid, FTS, galectin-1, galectin -7, JDP2, NF1, p53, Ras, Salirasib

## Abstract

Galectins are a family of β-galactoside-binding lectins that exert diverse extracellular and intracellular effects. Galectin-7 and galectin-1 show opposing effects on proliferation and survival in different cell types. Galectin-7 is a p53-induced gene and an enhancer of apoptosis, whereas galectin-1 induces tumorigenicity and resistance to apoptosis in several types of cancers. We show here that in cells derived from neurofibromin-deficient (*Nf1^−/−^*) malignant peripheral nerve sheath tumors (MPNSTs), Ras inhibition by S-*trans*,*trans*-farnesylthiosalicylic-acid (FTS; Salirasib) shifts the pattern of galectin expression. Whereas FTS decreased levels of both active Ras and galectin-1 expression, it dramatically increased both the mRNA and protein expression levels of galectin-7. Galectin-7 accumulation was mediated through JNK inhibition presumably resulting from the observed induction of p53, and was negatively regulated by the AP-1 inhibitor JDP2. Expression of galectin-7 by itself decreased Ras activation in ST88-14 cells and rendered them sensitive to apoptosis. This observed shift in galectin expression pattern together with the accompanying shift from cell proliferation to apoptosis represents a novel pattern of Ras inhibition by FTS. This seems likely to be an important phenomenon in view of the fact that both enhanced cell proliferation and defects of apoptosis constitute major hallmarks of human cancers and play a central role in the resistance of MPNSTs to anti-cancer treatments. These findings suggest that FTS, alone or in combination with chemotherapy agents, may be worth developing as a possible treatment for MPNSTs.

## INTRODUCTION

The Ras superfamily control many cellular functions including cell growth, differentiation, motility and survival [1-6] and play a major role in cell transformation. They alternate between a GDP-bound (inactive) and a GTP-bound (active) state through the action of guanine nucleotide exchange factors (RasGEFs) and GTPase activating proteins (RasGAPS). Active Ras was found to interact specifically with two members of the galectins family; galectin-1 and galectin-3. [7-9].

Galectins are a phylogenetically conserved family of lectins that share consensus amino-acid sequences and the carbohydrate recognition domain responsible for β-galactoside binding [10]. They are located within the cells (cytoplasm and nucleus) and are also secreted into the extracellular space. Although originally considered only as extracellular structural elements, a large body of evidence testifying to their role in intracellular signaling has accumulated over the last decade. Galectins have been implicated in several cellular processes, including apoptosis, cell survival, cell adhesion, immune response, and gene expression (reviewed in [11] and [12]).

Overexpression of galectin-1, a prototype member of this family, has been documented in many different tumor types [13-15], and in various aspects of tumor biology including migration and invasiveness, chemoresistance [16], angiogenesis [17], immune escape [18] and malignant progression [19-21]. Galectin-1 interacts with the small GTPases H-Ras-GTP in the plasma membrane, resulting in stabilization of H-Ras-GTP, clustering of H-Ras-GTP and galectin-1 in non-raft microdomains [22], subsequent binding to Raf-1 (but not to PI3K or Ral-GEF [7, 23]), activation of the ERK signaling pathway, and increased cell transformation [7].

As opposed to galectin-1, galectin-7—another member of the galectin superfamily—displays pro-apoptotic activity in various types of cells. Expression of galectin-7 is induced in the early steps of p53-mediated apoptosis and has been designated as the product of the p53-induced gene 1 (PIG1) [24]. A major function of p53 is control of apoptosis homeostasis [25], and it is the most frequently mutated gene in human tumors [25]. As opposed to galectin-7, galectin-1 is downregulated by p53 in glioma cells [26]. In line with its pro-apoptotic activity, galectin-7 inhibits DLD-1 cell proliferation *in vitro* and *in vivo* [27, 28] and is downregulated in transformed keratinocytes [29]. UVB irradiation induces apoptosis and galectin-7 expression in dependence with p53 [30, 31]. Ectopic expression of galectin-7 in HeLa and DLD-1 cells renders them more sensitive to a variety of apoptotic triggers, causes enhanced caspase-3 activity and poly(ADP-ribose) polymerase cleavage, and accelerated mitochondrial cytochrome-C release [32]. In addition, galectin-7 was found to bind directly to Bcl-2 in the mitochondria and to sensitize the mitochondria to apoptotic signals [33].

While galectin-7 negatively regulates some tumor types, it can stimulate the growth and/or development of others [34-37]. It thus seems that galectin-7 can act either as a positive or as a negative regulatory factor in tumor development, depending on the histological type of the tumor. Although the effect of p53 on galectin-7 expression is well established, little is known about how its transcription is regulated.

Although, as mentioned above, changes in expression levels of galectins have been implicated in many types of diseases including cancer, the role of galectins in neurofibromatosis type 1 (NF1) is still unknown. NF1 has an autosomal dominant mode of inheritance with a prevalence of about 1 in 3000 live births. It harbors a variety of phenotypes. The hallmark of NF1 is the neurofibroma, a benign peripheral nerve tumor comprised of transformed Schwann cells [38]. Neurofibromas undergo transformation into aggressive and chemotherapy-resistant malignant peripheral nerve sheath tumors (MPNSTs), which are prone to life-threatening metastasis [39].

Loss of neurofibromin Ras-GAP activity is associated with increased Ras-GTP and overactivation of Ras effectors [40], and reviewed in [41], leading to NF1 [42, 43]. The role of Ras in NF1-based malignancy suggests that Ras inhibitors such as *S*-*trans, trans*-farnesylthiosalicylic acid (FTS; Salirasib), which interfere with Ras-membrane anchorage [3], are likely to have useful therapeutic activity. Importantly, FTS interferes, both *in vitro* and *in vivo*, with the transformed phenotype of NF1-associated MPNST cell lines [44]. We recently discovered that FTS reverses the epithelial-mesenchymal (EMT)-like transition phenotype of NF1-deficient MPNST cells by perturbing the signaling of bone morphogenetic protein (BMP)4 and transforming growth factor (TGF)-β1 to SMAD-dependent and ERK-dependent pathways, inhibiting motility, spreading and gelatinase secretion, and alternating gene expression [45].

The activator protein-1(AP)-1 transcription-factor complex, which participates actively in cell proliferation, differentiation and cell transformation, is composed of homodimeric and heterodimeric complexes consisting of members of the Jun: Fos and Jun dimerization protein 2 (JDP2), activating transcription factors (ATFs) and other proteins [46]. c-jun, which is phosphorylated by c-Jun terminal kinase (JNK) through Ras-induced signaling [47-49], cooperates with Ras in cell transformation [50, 51] and has been shown to interfere with p53-induced apoptosis [52, 53]. JDP2 heterodimerizes with c-jun [54], and functions as a repressor of the AP-1 protein family by interfering with the c-jun-induced transformation [55]. JDP2 inhibits cell proliferation [56] and cell transformation, both induced by Ras [57]. Thus, on the one hand JDP2 inhibits cell transformation induced by Ras; on the other hand, it has been identified as a candidate oncogene in mouse hepatocellular carcinoma [58] and in a high-throughput screen in mice [59-61].

Here we show that Ras inhibition in NF1-deficient MPNST cells dramatically increases galectin-7 expression and decreases the expression of galectin-1. The increase in galectin-7 was dependent on JNK inhibition. We found that expression of galectin-7 itself modulates Ras signaling and renders MPNST cells more sensitive to apoptosis, suggesting the possible existence of cross-talk between Ras and galectin-7.

## RESULTS

### Ras inhibition induces galectin-7 and reduces galectin-1 expression in NF1-deficient MPNST cells

Ras inhibition by FTS in NF1-deficient MPNST cells inhibits their transformed phenotype both *in vitro* and *in vivo* [44], reverses their EMT-like phenotype, and alters gene expression [45]. One of the most significantly upregulated genes in our microarray analysis was the β-galactosidase-binding lectin, galectin-7, with an increase of 22.6-fold in its transcript in ST88-14 cells after FTS treatment. Galectin-7 is considered to be an apoptotic regulator, whose mRNA is highly induced by p53 [24] and whose expression sensitizes HeLa and DLD-1 cells to apoptosis through enhanced caspase-3 activity [32].

**Figure 1:**
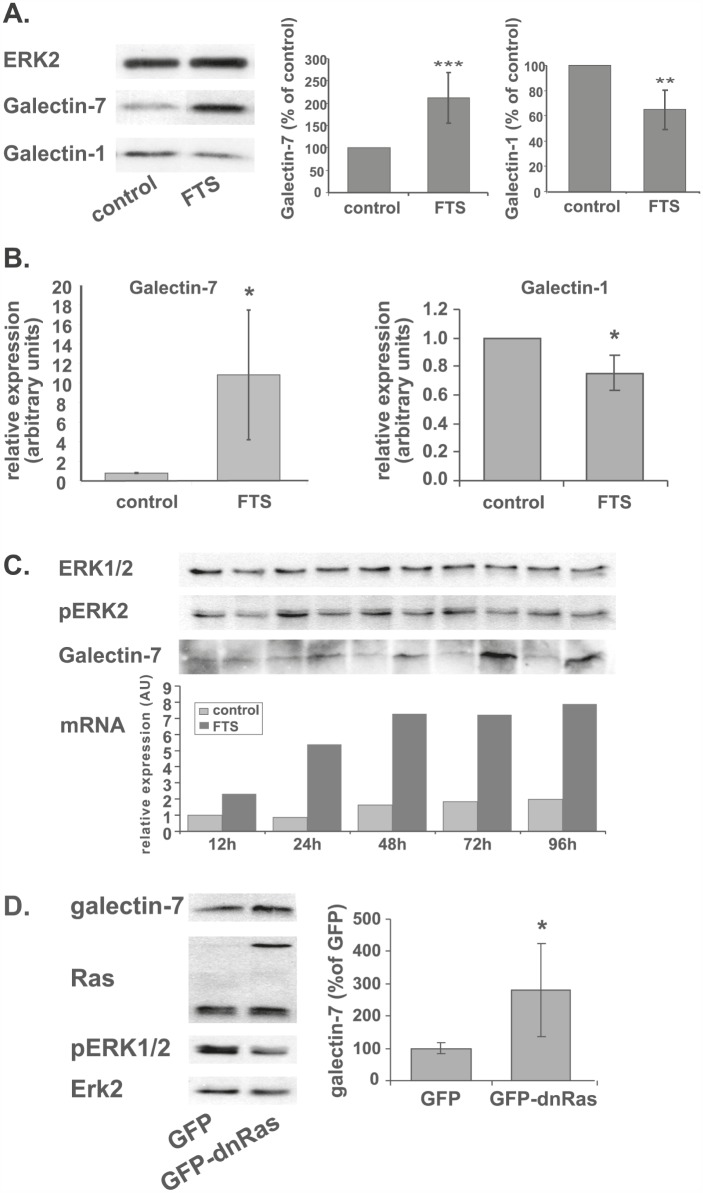
FTS induces a shift in galectin-1 and galectin-7 expression levels (A) ST88-14 cells were treated for 48 h with FTS (75 μM, 5% FCS) or vehicle followed by immunoblotting with galectin-7 or galectin-1 antibodies. ERK2 served as loading control. Immunoblots from a typical experiment are shown in the left panel. Graphs depict quantification of galectin-1 (right) and galectin-7 (center) (**p<0.01, ***p<0.001, n=6). (B) Relative expression of galectin-7 and galectin-1 mRNA were detected by real-time PCR (*p<0.005, n=4 and n=3 respectively). (C) ST88-14 cells were treated with FTS (75 μM, 5% FCS) for the indicated times and immunoblotted with anti-galectin-7, anti pERK 1/2 or anti ERK2 antibodies or subjected to real-time PCR for assay of galectin-7 mRNA. A typical experiment is shown. (D) ST88-14 cells were transfected with GFP or GFP-H-Ras(17N) (dnRas) by nucleofection, as described in Methods. After 24 h cells were lysed and subjected to western blot analysis with the indicated antibodies. Typical blots are shown in the left panel; right panel depicts quantification of galectin-7 (n=3, *p<0.05). ERK2 served as loading control.

Treatment with FTS (75 μM, 48 h, 5% serum) markedly increased the amounts of galectin-7 protein in the NF1-deficient MPNST cell lines ST88-14 (Figure 1A, 211% of control, p<0.001, n=6) and T265p21 (Supplementary Figure 1A). No such increase was seen after FTS treatment of the non-NF1 STS26T cell line or of the NF1-deficient cell line 90-8 (Supplementary Figure 1A). It is worth noting that both of the cell lines in which Ras inhibition induced an increase in galectin-7 (i.e., ST88-14 and T265P21) harbor wild-type p53, whereas the two cell lines in which galectin-7 was unaffected by FTS harbor mutated p53 [66]. FTS treatment of ST88-14 cells also increased their galectin-7 mRNA content, as detected by real-time PCR (Figure 1B, 1040% of control, p<0.05, n=4). In contrast to galectin-7 mRNA, our microarray analysis indicated that galectin-1 mRNA was downregulated by 14 fold following FTS treatment, a trend that was validated by real-time PCR analysis (Figure 1B, 76% of control, p<0.05, n=4) and was accompanied by a decrease in galectin-1 protein in ST88-14 cells (Figure 1A, 64% of control, p<0.01, n=4). These results are consistent with earlier reports of galectin-1 downregulation by inhibition of Ras [7, 8].

To follow the dynamics of the FTS-induced increase in galectin-7 mRNA and protein, we treated ST88-14 cells with FTS (75 μM, 5% FCS), and monitored the change over time by real-time PCR and Western blotting for different time periods From 12 h after FTS treatment galectin-7 mRNA increased in a time-dependent manner, reaching a plateau at 48 h after treatment. Galectin-7 protein levels started to increase at 24 h after treatment and reached a maximum at 72 h (Figure 1C).

To determine whether the FTS-induced upregulation in galectin-7 protein was a result of the Ras inhibition, we evaluated galectin-7 in ST88-14 and T265P21 cells transfected with GFP-H-Ras(17N) (dnRas) or, as a control, with GFP. Transfection of dnRas induced a significant increase in galectin-7 expression levels in both the ST88-14 (Figure 1D, 280% of GFP, p<0.05, n=4) and the T265p21 cells (Supplementary Figure 1B, 122% of GFP, p<0.06, n=3). As expected, the cells transfected with dnRas exhibited decreased ERK phosphorylation, indicating that the transfected vector was indeed reducing Ras activation (Figure 1D). This strengthens the notion that the FTS-induced increase in galectin-7 is a result of Ras inhibition.

The protein synthesis inhibitor cycloheximide completely inhibited the FTS-induced increase in galectin-7 (Supplementary Figure 1C), further supporting the notion that regulation of the transcription was mediated by Ras.

### Galectin-7 expression is induced by JNK inhibition

**Figure 2:**
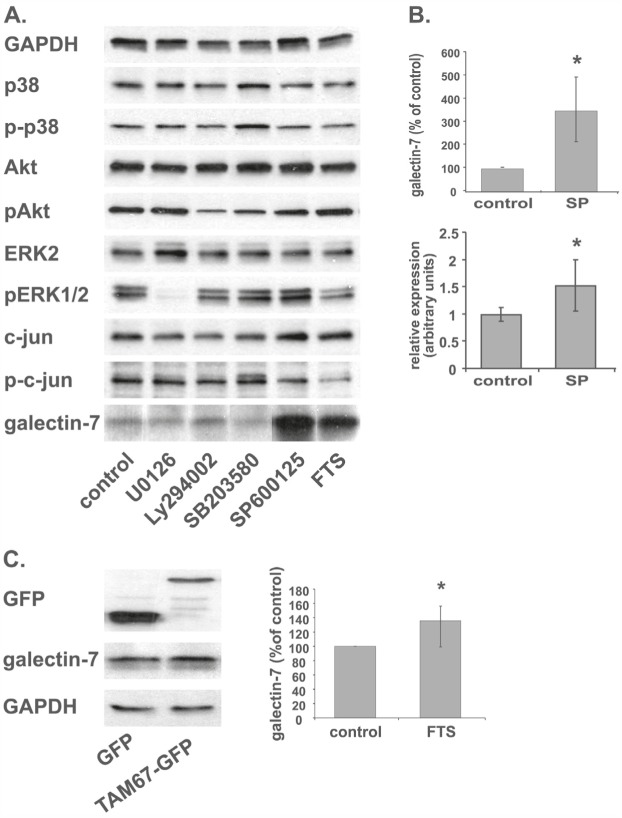
JNK inhibition increases galectin-7 protein and mRNA levels (A) ST88-14 cells were treated for 48 h with U0126 (10 μM), LY294002 (20 μM), SB203580 (4 μM), SP600125 (25 mM), FTS (75 μM) or vehicle, followed by immunoblotting with the indicated antibodies. (B) Quantification of ST88-14 treated as in (A) and assayed for galectin-7 protein by western blotting (upper panel) or for mRNA by real-time PCR (lower panel). SP for SP600125 (C) ST88-14 cells were transfected with GFP or GFP-TMA67 by nucleofection, as described in Methods. After 24 h, cells were lysed and subjected to western blot analysis with the indicated antibodies. Typical blots are shown in the left panel; right panel depicts quantification of galectin-7 protein expression (n=3, *p<0.05). GAPDH served as loading control.

To find out which Ras-signaling pathway leads to the galectin-7 upregulation described above, we employed several chemical inhibitors of some of the most prominent Ras effectors: U0126, a MEK inhibitor; LY 294002, a PI3K inhibitor; SB203580, a p38 inhibitor, and SP600125, a JNK inhibitor. Western blot analysis showed that phosphorylation of all the relevant target proteins was curbed by their respective inhibitors (Figure 2A), with the exception of p38, on which SB203580 had no effect. Notably, in ST88-14 cells inhibition of the JNK pathway alone induced a pronounced increase in galectin-7 protein (Figure 2A, B), which was accompanied by an increase in galectin-7 mRNA (Figure 2B). Treatment of T265p21 cells with SP600125 also resulted in a robust increase in galectin-7, like that in ST88-14 cells (Supplementary Figure 2A).

By using a dominant-negative c-jun protein (TAM67), we further verified that inhibition of JNK and its phosphorylation target c-jun indeed induced galectin-7 protein. Transient transfection with TAM67 significantly increased galectin-7 expression in ST88-14 cells, strengthening the notion that c-jun is a major player in the Ras-induced increase in galectin-7.

### Ras inhibition induces galectin-7 transcription through p53, c-jun, and JDP2

**Figure 3:**
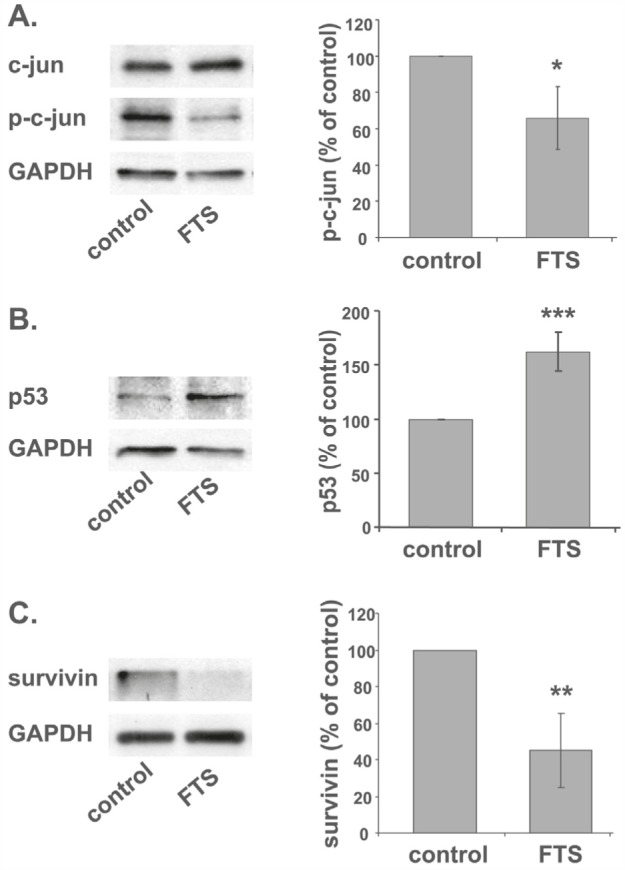
Ras inhibition induces galectin-7 transcription through p53 and c-jun (A, B) ST88-14 cells were treated for 48 h with FTS (75 μM, 5% FCS) or vehicle followed by immunoblotting with anti-p-c-jun and total c-jun (A) or anti-p53 (B) antibodies. GAPDH served as loading control. Immunoblots from a typical experiments are shown in the left panels; graphs depicting quantification{?depicting assay} of p-c-jun (A) or p53 (B) are shown in the right panel (A; *p<0.05, n = 3, B; ***p<0.001, n = 5).

Following the notion that galectin-7 accumulates in response to inhibition of Ras or c-jun, we examined whether Ras inhibition in ST88-14 cells directly inhibits c-jun. A decrease in phospho-c-jun in these cells was indeed observed after treatment with FTS (Figure 3A, 67% of control, *p<0.05, n=3), suggesting that Ras inhibition induces galectin-7 expression by inhibiting c-jun activation.

Expression of galectin-7 is regulated by the tumor suppressor p53 [24], which is one of the most frequently mutated genes in cancer [25]. Analysis of the promoter region of *galectin-7* gene using a Genomatix Genome Analyzer (Genomatix Software GmbH [65]) indeed revealed a putative p53-binding site (Supplementary Figure 2B). ST88-14 cells harbor wild-type p53, although its levels under normal growth conditions are small. We therefore followed changes in the amounts of p53 protein after FTS-induced inhibition of Ras in ST88-14 and T265P21 cells. In agreement with results obtained in the case of colon cancer [67], treatment with FTS resulted in a significant accumulation of p53 protein (ST88: Figure 3B, 163% of control, ***p<0.001, n=5; T265P21: Supplementary Figure 2C, 213% of control, **p<0.01, n=3).

Upregulation of p53 in ST88-14 cells was also found here to lead to a significant decrease in the anti-apoptotic protein survivin (Fig 3C, 45% of control, p<0.01, n=5) and in its mRNA (Figure 3C, 8% of control, p<0.001, n=4). This was an interesting finding, in view of a report that transcription of survivin is repressed by wild-type p53 [68, 69]. Our finding is also in line with the previously described decrease in survivin by FTS in U87 [70] and DLD1 cells [71].

**Figure 4:**
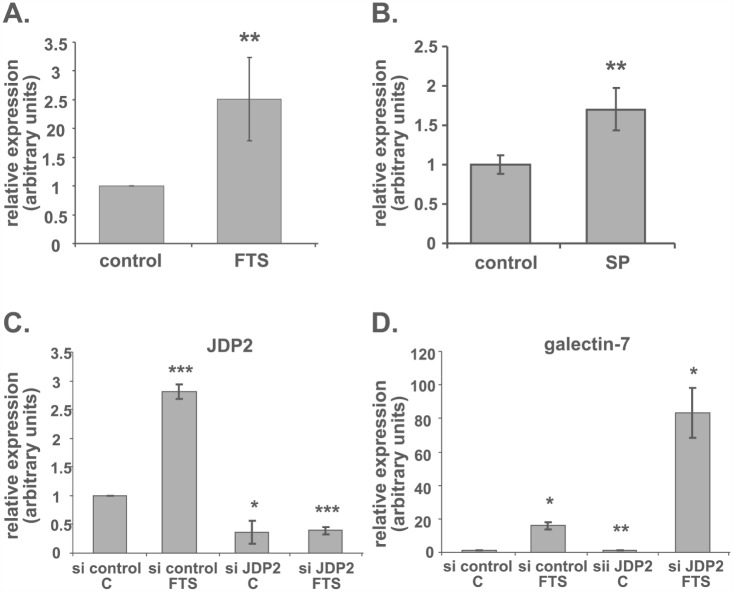
JDP2 inhibition boosts galectin-7 transcription (A, B) ST88-14 cells were treated for 48 h in 5% FCS with FTS (A; 75 μM) or with SP600125 (B; 25 mM). JDP2 mRNA was assayed by real-time PCR (**p<0.01; (A), n=6; (B), n=3). (C, D) ST88-14 cells transfected with non-targeting siRNA (si control) or with siRNA against JDP2 (si JDP2) were treated, 24 h after transfection, with FTS (75 μM, 5% FCS) or vehicle. JDP2 (C) and galectin-7 (D) mRNA were assayed by real-time PCR (*p<0.05, **p<0.01, ***p<0.001, n=3).

Given our observations that JNK inhibition induced a dramatic increase in galectin-7 and that FTS itself inhibits c-jun phosphorylation, we searched for c-jun regulatory elements in the galectin-7 promoter. Using Genomatix, we found putative binding sites for AP-1 and for the AP-1 inhibitor JDP2 (Supplementary Figure 2B). Because JDP2 participates in the p53 signaling pathway [72] and is regulated by c-jun [73], we investigated its possible involvement in the regulation of galectin-7 transcription. First, we analyzed changes in the amounts of JDP2 mRNA after FTS treatment. Figure 4A shows that JDP2 transcripts were increased by 2.5-fold in FTS-treated cells compared to control (**p<0.01, n=6). Inhibition of JNK by SP600125 also increased JDP2 mRNA (Figure 4B, 1.7-fold compared to control, ** p<0.01, n=3). Other studies demonstrated the phosphorylation of JDP2 on Thr148 by JNK leading the protein to proteasomal degradation [73], supporting the negative role of JNK on JDP2 levels.

Next, we examined effect of using a specific small interfering RNA (siRNA) to decrease JDP2 expression. Figure 4C shows that transfection of cells with a specific siRNA against JDP2 reduced JDP expression to 0.36-fold of that obtained with a nonspecific siRNA (*p<0.05, n=3). In the latter transfected cells, the FTS-induced increase in JDP2 was maintained (Figure 4C, 2.8-fold of control; ***p<0.001, n=3), exactly as shown in Figure 1A. However, JDP2-siRNA markedly decreased the FTS-induced JDP2 accumulation to 0.14-fold of that in the non-target-siRNA transfected cells treated with FTS. Surprisingly, after the former transfection, induction of galectin-7 mRNA in the FTS-treated cells showed a dramatic 83.3-fold increase compared to an increase of only 5.2-fold in the control FTS-treated cells transfected with non-target siRNA (Figure 4D). Accumulation of galectin-7 mRNA was also significantly increased without FTS treatment: in cells transfected with siRNA for JDP2 and treated with vehicle, galectin-7 mRNA was 1.46-fold higher than in the nonspecific siRNA transfectants (Figure 4D, **p<0.01, n=3).

### Galectin-7 expression inhibits Ras and sensitizes ST88-14 cells to apoptosis

**Figure 5:**
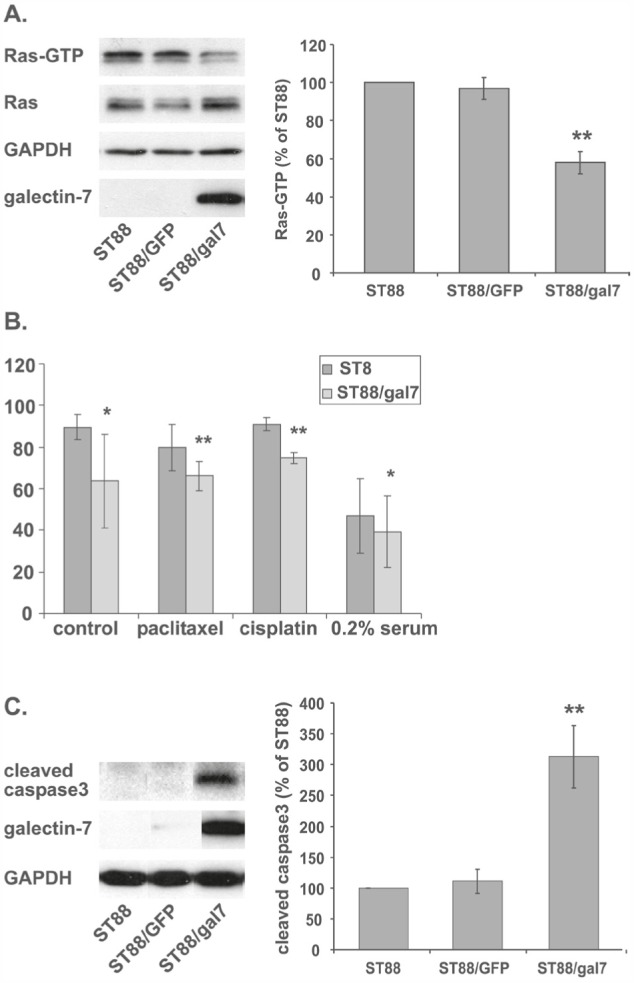
Galectin-7 expression inhibits Ras and sensitizes ST88-14 cells to apoptosis (A) Wild-type ST88-14 cells (ST88) or ST88-14 cells stably infected with GFP (ST88/GFP) or galectin-7 (ST88/gal7) were subjected to Ras-GTP pull-down assay and immunoblotted with anti-Ras, anti-galectin-7, or anti-GAPDH antibodies. Left panel depicts a typical experiment; quantification (% of control) is shown in the right panel (** p<0.01, n=3). (B) ST88 or ST88/gal7 cells were treated with paclitaxel (10 nM), cisplatin (1 μM), or vehicle in growth medium or under serum deprivation (0.2% FCS) for 24 h, then collected, stained with FITC-conjugated annexin V and PI, and analyzed by flow cytometry (*p<0.05, **p<0.01, n=3). (C) ST88, ST88/GFP or ST88/gal7 cells (5% FCS, 48 h) were lysed and immunoblotted using cleaved caspase 3, galectin-7 or GAPDH antibodies. Typical immunoblots are shown in the left panel and quantification of cleaved caspase 3 (% of control; **p<0.01, n=4) in the right panel.

We have previously shown that galectin-1 expression stabilizes Ras in its GTP-binding state and that antisense galectin-1 reduces Ras activation [7]. Having detected an increase in galectin-7 and a decrease in galectin-1 in ST88-14 cells after Ras inhibition, we wanted to find out whether expression of galectin-7 affects Ras activation. To that end, ST88-14 cells were infected with viruses containing galectin-7 DNA (ST88/gal7) or, as a control, GFP (ST88/GFP). After selection and validation of expression, the amounts of Ras-GTP in the ST88/gal7 cells were compared to those in ST88/GFP and in noninfected ST88-14 cells. Notably, stable expression of galectin-7 in ST88-14 cells resulted in a marked reduction of Ras activity to 58% of that in GFP-infected or noninfected cells (Figure 5A, **p<0.01, n=3). We could not detect any significant changes in pERK, p-Akt or p-c-jun levels (data not shown).

That galectin-7 expression sensitizes cells to caspase-mediated apoptosis induced by several agents has by now been well established [32]. Unpublished data from our lab has demonstrated that treatment of ST88-14 cells with FTS together with several chemotherapy agents. i.e., paclitaxel, doxorubicin, or cisplatin induces a synergistic effect on cell proliferation and enhances cell death. We therefore examined whether the FTS-induced accumulation of galectin-7 might render NF1-deficient cells more sensitive to apoptosis. ST88-14 and ST88/gal 7 cells were treated with different cell-death inducers (paclitaxel, cisplatin, or serum deprivation (0.2% FCS)) and assayed for cell viability using annexin-V/PI staining followed by FACS analysis. As shown in Figure 5B, expression of galectin-7 reduced the percentage of live (nonapoptotic) cells as indicated by negative staining for both PI and annexin-V. Those results indicate that galectin-7 expression sensitizes ST88-14 cells to apoptotic triggers, in agreement with the report by Kuwabara et al. [32].

Next, we examined the nature of galectin-7-induced susceptibility to apoptosis. Galectin-7 expression has been shown to cause enhanced caspase-3 activity [14, 32] and to sensitize urothelial cancers to cisplatin [28]. In agreement with these findings we detected high levels of cleaved caspase-3 in ST88/gal7 cells, whereas in control cell lines the cleaved form of caspase-3 was undetectable (Figure 5C, **p<0.01, n=4).

## DISCUSSION

We show here that in the NF1-deficient ST88-14 and T265P21 MPNST cell lines, Ras inhibition by FTS or dnRas dramatically induced expression of the pro-apoptotic protein galectin-7 (Figure 1 for ST88-14, Supplementary Figure 1B for T265P21). This was evident (for ST88-14) from the microarray analysis (22.6-fold increase), RT‒PCR (1040% of control), and western blotting (211% of control). In contrast, FTS treatment reduced the expression of galectin-1, as also shown by microarray analysis, real-time PCR, and western blot analysis (Figure 1A, B). The negative regulation of Ras on galectin-7 expression is probably mediated through the consequent activation of the JNK‒;c-jun pathway, because the inhibition of JNK by SP600125 or dn‒c-jun, but not inhibitors of other Ras signaling pathways, reproduced the FTS-induced increase in galectin-7 protein seen in ST88-14 and T265P21 cells (Figure 2A‒C and Supplementary Figure 2A). Moreover, FTS treatment itself inhibited c-jun phosphorylation (Figure 3A).

Bioinformatics analysis of galectin-7 upstream elements revealed predicted AP-1/c-jun and p53 regulatory elements. The role of p53 in galectin-7 induction has been well established [24, 26, 30] [31]. In conformity with this, the FTS-induced increase in galectin-7 was accompanied by an increase in the amounts of p53 protein (Figure 3B). Because galectin-7 was found to increase after Ras inhibition, it is reasonable to suggest that the regulation of this increase might be mediated by a c-jun-related repressor such as JDP2. This protein binds members of the AP-1 family such as c-jun, ATF-3, and ATF-2, preventing them from exerting their transcription activity [74]. The galectin-7 promoter also contains a potential JDP2-binding element. Accordingly, inhibition of JDP expression by siRNA transfection resulted in a dramatic increase in galectin-7 mRNA after FTS treatment (a 5.2-fold increase compared to FTS-treated control siRNA-transfected cells, and an increase of 83-fold compared to vehicle-treated control si-RNA transfected cells (Figure 4C, D). Unexpectedly, we also detected an unexplained increase in JDP2 mRNA after inhibition of Ras or JNK (Figure 4A, B).

Galectin-7 expression has been shown to sensitize cells to apoptosis and enhance cleavage of caspase-3 [32, 33]. Importantly, stable expression of galectin-7 in ST88-14 cells rendered them more sensitive to apoptotic signals and induced caspase-3 cleavage under normal growth conditions (Figure 5B, C). Interestingly, active Ras in ST88-14 cells stably expressing galectin-7 was reduced in comparison to that in naȉve or GFP-expressing cells (Figure 5A).

Studies have shown that expression of galectin-7 is induced as a result of cellular stress such as UV irradiation [30, 31] and is mediated by p53 [24, 75]. We report here, for the first time, that the expression of galectin-7 is negatively regulated by Ras-GTP. In the MPNSTs of NF1 patients, lack of functional neurofibromin leads to chronically active Ras. Inhibition of Ras in MPNST cells in the present study led to dramatic expression of galectin-7, mediated most probably through the FTS-induced decrease in p-c-jun levels, since inhibition of JNK or c-jun yielded similar induction of galectin-7. Notably, in two p53-mutated MPNST cell lines, STS26T and 90-8 [66], no increase in galectin-7 after Ras inhibition was detectable. It thus seems that p53 is a key factor in galectin-7 expression. Moreover, FTS-induced inhibition of Ras significantly increased p53 expression and the consequent reduction of survivin in the wild-type p53 cell lines. Induction of p53 after Ras inhibition has been reported in colon cancer [76] and in pancreatic cancer [67] cell lines. Inhibition of c-jun was shown to promote apoptosis, arguably through a p53-dependent mechanism, and to reduce liver cancer in mice [52].

We postulate that in NF1-deficient MPNST cells, high Ras activity results in low p53 levels together with intense c-jun phosphorylation. P-c-jun then heterodimerize with JDP2 to inhibit the galectin-7 promoter. Ras inhibition induced by FTS treatment increases p53 levels, thereby triggering p53-dependent galectin-7 transcription. The FTS-induced Ras inhibition also inhibits c-jun phosphorylation, thus dispersing the c-jun/JDP2 heterodimer and allowing galectin-7 transcription. Indeed, decreasing JDP2 levels using specific siRNA might also disperse the dimer thereby freeing the *gal7* promoter and making the cells more sensitive to FTS-induced galectin-7 accumulation. High galectin-7 levels in turn rendered MPNST cells more sensitive to apoptotic factors, promoted the apoptosis process and restrained Ras activity, thus sensitizing the cells to chemotherapy or radiotherapy. In addition, the secreted galectin-7 might be taken up by neighboring cells and affect their Ras signaling, as previously reported for galectin-1 [77]. Moreover, we were able to demonstrate a possible reciprocity in galectin-7/Ras relations, since galectin-7 itself reduced Ras activity. This is reminiscent of the effect of galectin-1 on stabilizing Ras in its GTP-binding state [7].

Galectin-1 promotes tumor growth [15, 19-21] and has been described as a cytokine-like factor that participates in the promotion of tumor angiogenesis [17]. Moreover, Ras can be dislodged from the plasma membrane by inhibition of galectin-1 employing anti-sense DNA [7], by application of the anti-angiogenic peptide anginex [77], or by disruption (via a point mutation (11A) [78]) of its ability to bind Ras. This results in a decrease in Ras-GTP and inhibition of the Raf-MEK-ERK cascade [7, 77, 78]. Previously we indeed observed that anginex significantly inhibits proliferation of NF1-deficient MPNST cells (unpublished data). Thus, Ras-GTP might cooperate with galectin-1 to induce tumorigenicity in NF1-deficient cells, while “shutting down” galectin-7 expression.

**Figure 6:**
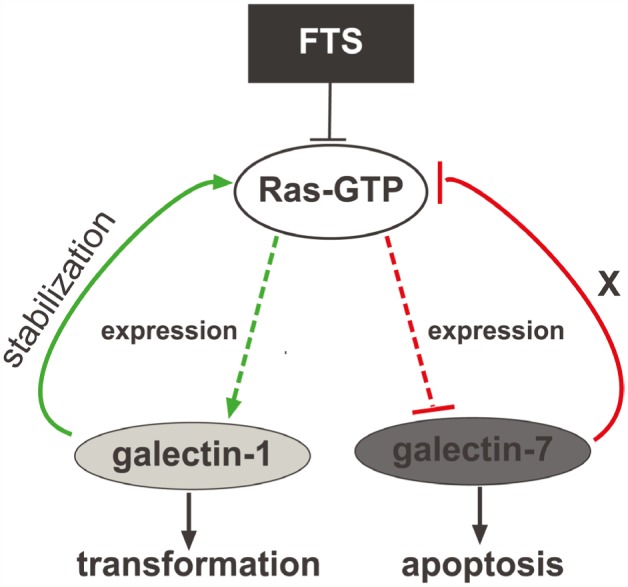
Model depicting the galectin-1/galectin-7 switch High Ras-GTP increases galectin-1 expression and inhibits galectin-7 expression through JNK. Galectin-1 itself stabilizes and increases Ras-GTP, thereby inducing transformation. Inhibition of Ras by FTS or dnRas reduces galectin-1 expression and induces robust expression of galectin-7. High levels of galectin-7 inhibit Ras activation and sensitize cells to apoptosis.

Altogether, these results point to a galectin-1/galectin-7 switch, in which FTS-induced Ras inhibition reverses the expression pattern of MPNST galectins, as suggested in the model in Figure 6. Galectin-7 reportedly has some effects that oppose those of galectin-1: whereas galectin-1 promotes tumor growth [15, 19-21] and induces resistance to apoptosis, galectin-7 induces apoptosis in several types of cancer cells [28, 32]. It is tempting to speculate on the presence of a mechanism of inhibitory cross-talk in which in several transformed cells, and particularly in NF1-deficient MPNSTs, high Ras activity stimulates galectin-1 and inhibits galectin-7 expression. Inhibition of Ras-GTP by FTS decreases galectin-1 expression, promoting inhibition of cell transformation while increasing galectin-7 expression with resulting heightened sensitivity to apoptosis.

## MATERIALS AND METHODS

### Cell culture

The human MPNST cell lines ST88-14, T265P21, 90-8 (NF1^−/−^) and the STS26T cell line (NF1^+/+^) were a kind gift from Dr. Nancy Ratner (Cincinnati Children’s Hospital and Medical Center, University of Cincinnati). All cell lines were routinely checked for mycoplasma, maintained, and subjected to genetic analysis, as described earlier [44]. Cells were plated at 0.75 × 10^6^ cells per 10-cm dish or 20 × 10^4^ cells per 6-well plate.

### Materials

FTS was a kind gift from Concordia Pharmaceuticals. Cycloheximide was from Sigma-Aldrich, (St. Louis, MO) and LY294002, U0126, SB203580, and SP600125 were from Calbiochem (San Diego, CA). The antibodies used were: pERK (Sigma-Aldrich); ERK, p-c-jun (Serine 63), c-jun (Santa Cruz Biotechnology, Santa Cruz, CA); pAkt, Akt, p-p38 (Thr180/Tyr182), p38, survivin, and GAPDH (Cell Signaling Technology, Danvers, MA); galectin-1 (PeproTech, Rocky Hill, NJ); galectin-7 (Abcam, Cambridge, MA); pan-Ras (Calbiochem); green fluorescent protein (GFP); naȉve goat IgG, horeseradish peroxidase-conjugated goat anti-mouse IgG or goat anti-rabbit IgG were from Jackson ImmunoResearch Laboratories (West Grove, PA).

The following plasmids were used: PEF1 and PEF1-galectin-7 were the kind gift of Fu-Tong Liu [32]. pEGP-TAM67 (TAM67) [62] was a kind gift from Prof. Lily Vardimon (Department of Biochemistry, Tel Aviv University). pBabe-Gal7 was generated by amplifying galectin-7 with primers containing the restriction enzymes HindIII (forward) and KpnI (reverse), cloned into PGEM®-T-Easy (Promega), then digested with BamHI/SalI and cloned into pBabe-Puro digested with BamHI/SalI.

Protein bands were quantified by densitometry with Image EZQuant-Gel software (EZQuant Ltd., Tel Aviv, Israel).

### Ras-GTP assays

Lysates containing 0.5 mg protein were used to measure Ras-GTP by the glutathione S-transferase fused to the Ras-binding domain of Raf (GST-RBD) pull-down assay as described [63]. The lysates were then western-blotted with pan-Ras Ab, as described above.

### RT‒PCR analysis

Extracts of total RNA (1 μg) were reverse-transcribed in a total volume of 20 μl using the Verso− RT–PCR Kit (Thermo Scientific, Rockford IL, USA) according to the manufacturer’s instructions. cDNA samples (1 μg) were used for RT–PCR (QPCR SYBR® Green Mix Plus ROX Vial; ABgene, [Epsom, UK]).

The primers used were:

**Table:** 

GUSB:	CTCATTTGGAATTTTGCCGATT CCGAGTGAAGATCCCCTTTTTA
Galectin-1:	CTCTCGGGTGGAGTCTTCTG CCGAGTGAAGATCCCCTTTTTA
Galectin-7:	ATGTCCAACGTCCCCCACAAG TGACGCGATGATGAGCACCTC
JDP2:	CGCCGGGAGAAGAACAAAG GGATTCCCGCTGCAGAAAC

GUSB was used as a reference gene for normalization of relative mRNA expression.

### Fluorescence-activated cell sorter analysis

ST88-14 cells seeded in 6-well plates were treated as described in figure legends, and were then collected and washed with phosphate-buffered saline (PBS). Cells were subjected to Annexin-V-PI kit according to manufacturer’s instructions (BD biosciences, Franklin Lakes, NJ USA) and analyzed by fluorescence-activated cell sorter (FACS; FACSCalibur, Becton Dickinson, Los Angeles, CA). Data were analyzed by FlowJo data analysis software package (TreeStar, Ashland, OR).

### Transfections

Plasmid transfection: Transfections with GFP-Ras(17N), pEGP-TAM67 or GFP (10^6^ cells, 2 μg DNA; Ingenio Electroporation, Mirus, Madison, WI) were carried out by electroporation using Amaxa® (Lonza, Basel, Switzerland).

Small interfering RNA (siRNA) transfection: ST88-14 cells were plated 24 h prior to transfection (10^4^ cells per well in 12-well plates) and transfected with 50 nM ON-TARGETplus JDP2 siRNA oligos as well as ON-TARGETplus siCONTROL nontargeting pool, using DharmaFECT 1 Transfection Reagent (Thermo Fisher Scientific) according to the manufacturer’s instructions. As an indicator of transfected cells, we used the siGLO Green transfection indicator (Thermo Scientific), followed by treatments as described in the figure legends.

### Infections

Retroviruses were produced by standard protocols, as described [64]. Infected cells were allowed to recover and were selected by puromycin (1 μg/ml). Expression was validated by western blotting and fluorescence microscopy. Cells were maintained in growth medium containing 0.5 μg/ml puromycin, and subjected to routine analysis.

### Statistical calculations

Significant differences between the results obtained by the experimental and the control groups in each experiment were determined by paired or unpaired Student’s *t* test, as appropriate, using Microsoft Excel. P-values that were equal to or smaller than 0.05 were considered significant. Data are presented as means ± standard error of the mean (SEM). Statistical significance at *p*-values of * ≤ 0.05, ** ≤ 0.01 and *** ≤ 0.001 are indicated.

### Image processing

Adobe Photoshop was employed to adjust for brightness/contrast or cropping of images. The promoter region of galectin-7 was analyzed using Genomatix Genome Analyzer (Genomatix Software GmbH [65]).

## Supplementary Figures


