# Supplementary material for: Ras inhibition boosts galectin-7 at the expense of galectin-1 to sensitize cells to apoptosis
Source: Oncotarget. 2013 Feb 24;4(2):256–68. doi: 10.18632/oncotarget.844 (PMC3712571; doi:10.18632/oncotarget.844)
Supplement: Supplementary file 1 [file oncotarget-04-256-s001.pdf]

Ras inhibition boosts galectin-7 at the expense of galectin-1 to sensitize cells to apoptosis - Barkan et al

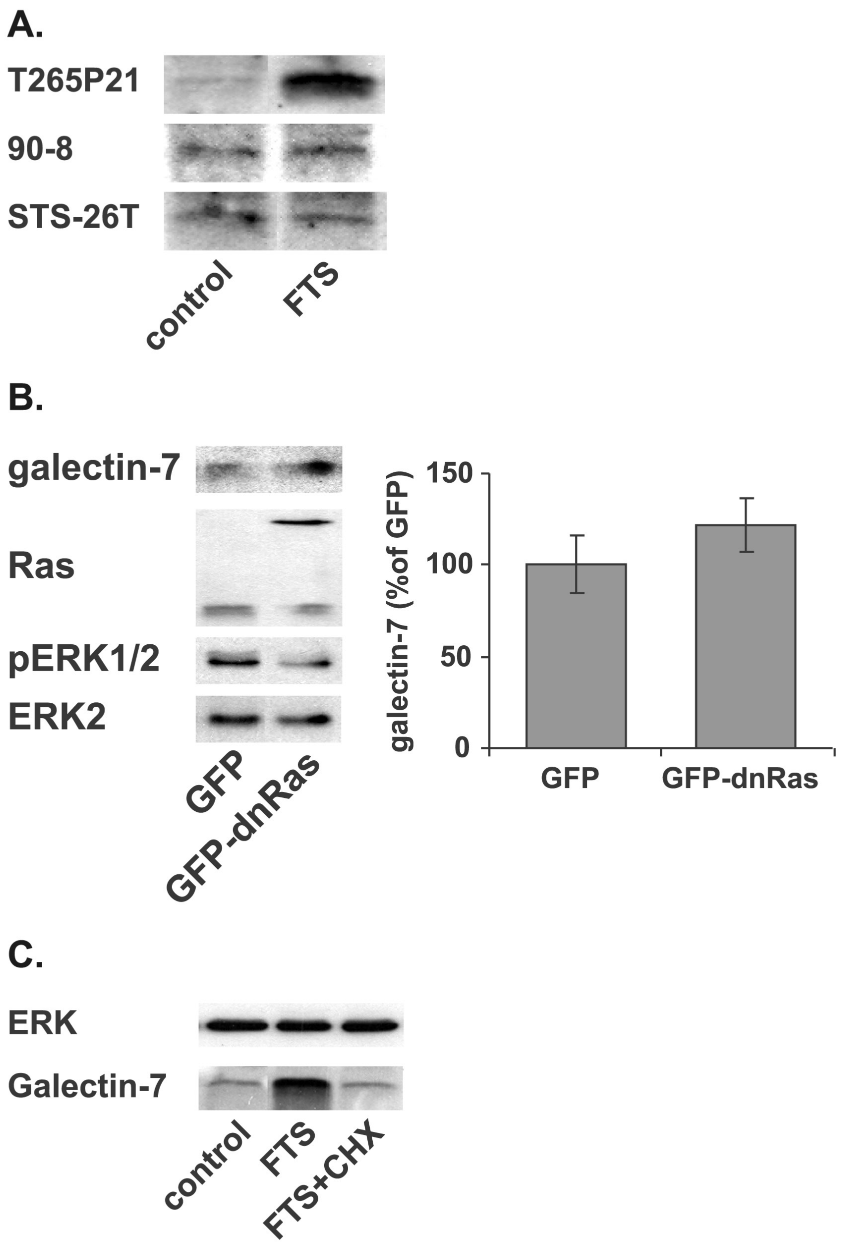

Supplementary Figure 1: (A) T265P21, 90-8 or STS26 cells were treated for 48 h with FTS (75 µM, 5% FCS) or vehicle and then immunoblotted with anti-galectin-7 antibody. A typical experiment is shown. (B) T265P21 cells were transfected with GFP-H-Ras(17N) or with GFP alone, by nucleofection, as described in Methods. After 24 h the cells were lysed and subjected to western blot analysis with the indicated antibodies. Typical blots are shown in the left panel; the right panel depicts quantification of galectin-7 ($p=0.06$, $n=3$). ERK2 served as loading control. (C) ST88-14 cells were treated for 48 h in 5% FCS with FTS (75 µM), cycloheximide (6.25 µM) or vehicle and then immunoblotted with galectin-7 or ERK2 antibodies.

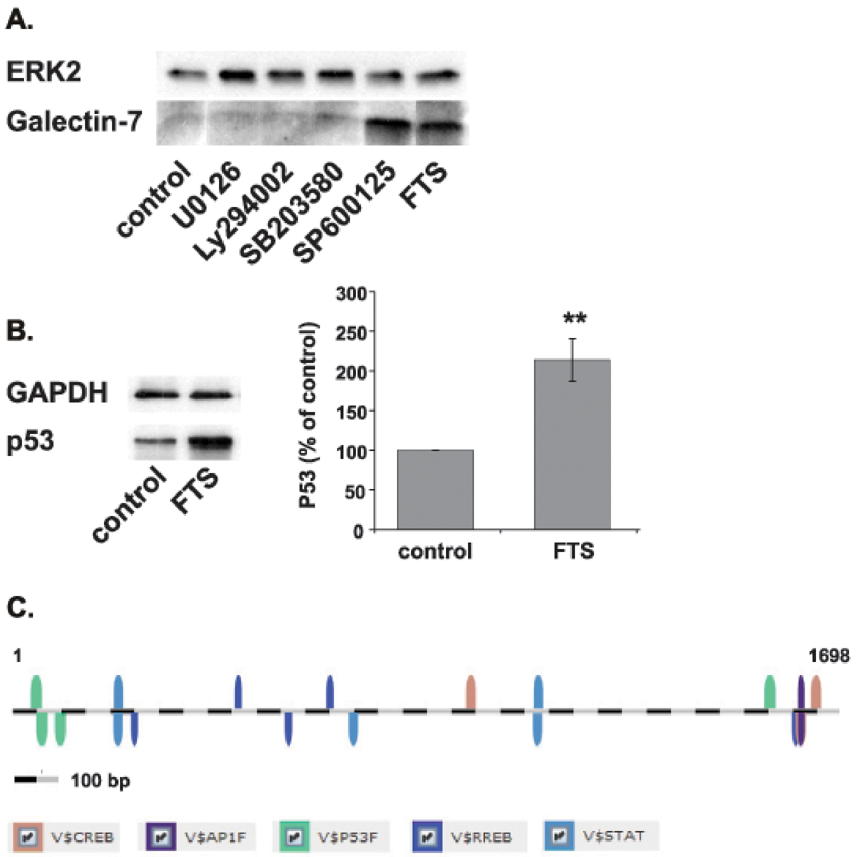

Supplementary Figure 2: (A) T265P21 cells were treated for 48 h with U0126 (10 µM), LY294002 (20 µM), SB203580 (4 µM), SP600125 (25 mM), FTS (75 µM) or vehicle and then immunoblotted with the indicated antibodies. (B) Analyses of galectin-7 promoter region using Genomatix software. Selected transcription factors are shown. (C) Immunoblots from a typical experiments are shown in the right panels; graphs depicting quantification of p53 are shown in the left panel (**p<0.01, n=4).
